# Supplementary material for: Novel Macromolecular and Biobased Flame Retardants Based on Cellulose Esters and Phosphorylated Sugar Alcohols
Source: Polymers (Basel). 2023 Jul 27;15(15):3195. doi: 10.3390/polym15153195 (PMC10420857; doi:10.3390/polym15153195)
Supplement: Supplementary file 1 [file polymers-15-03195-s001.zip › polymers-2493805-supplementary.pdf]

Supporting Information

Novel macromolecular and biobased flame-retardants based on cellulose esters and phosphorylated sugar alcohols

Matay Kaplan [1], Michael Ciesielski [1*], Sabine Fuchs [2], Christoffer Getterle [2], Frank Schönberger [1] and Rudolf Pfaendner [1]

[1] Fraunhofer Institute for Structural Durability and System Reliability LBF
[2] Hamm-Lippstadt University of Applied Sciences
* Correspondence: michael.ciesielski@lbf.fraunhofer.de

Abstract: The increasing demand to provide sustainably produced plastic materials requires a.o., the development of biobased flame-retardants (FRs) for applications where flame retardancy is essential. To meet those challenging new sustainability requirements, a set of novel phospho-rus-containing cellulose esters were synthesized by an efficient two-step procedure. In a first step cellulose was treated with acrylic anhydride to synthesize acrylate-functionalized cellulose esters – more specifically: cellulose acrylate butyrate (CeAcBu) and propionate (CeAcPr). Subse-quently, phosphorylated anhydro erythritol (PAHE), synthesized from the sugar alcohol erythri-tol, was added to the acrylate-functionalized cellulose esters via Phospha-Michael addition. For comparison a cellulose ester based on 6H Dibenzo[c,e][1,2]oxaphosphorin-6-on (DOPO) was prepared analogously. The acrylate-functionalized cellulose esters and novel FRs were charac-terized by NMR spectroscopy. TGA investigations of PAHE-functionalized CeAcBu revealed an onset temperature of decomposition (2 % mass loss) of approx. 290 °C. The novel PAHE-based FR was incorporated into a polypropylene-polyethylene copolymer (PP-co-PE) together with poly-tert-butylphenol disulfide (PBDS) (8 wt% / 2 wt%) as synergist. The PP-PE samples achieved V2 classification in the UL 94 V test. In addition, specimens of a rapeseed oil-based polyamide containing PAHE-functionalized CeAcBu at 20 wt% loading yielded a V2 rating with short burning times.

Keywords: flame retardant; biobased; sugar alcohol; acrylic anhydride; cellulose acrylate; esterification; Phospha-Michael addition; polypropylene-polyethylene copolymer

Table of content

I. ^{1}H and ^{31}P NMR Spectra of synthesized components 3

Cellulose acrylate propionate (4-CeAcPr) 3

Phosphorylated cellulose acrylate propionate with PAHE (6-CeAcPr-PAHE) 4

Cellulose acrylate propionate (4-CeAcPr^{SA}) 5

Phosphorylated cellulose acrylate propionate with PAHE (6-CeAcPr^{SA}-PAHE) 6

Phosphorylated cellulose acrylate propionate with DOPO (6-CeAcPr-DOPO) 7

Phosphorylated cellulose acrylate butyrate with DOPO (6-CeAcBu-DOPO) 8

II. Correlation spectroscopy (COSY) 9

COSY of 4-CeAcBu 9

COSY of 6-CeAcBu-PAHE 10

III. Heteronuclear single quantum coherence spectroscopy (HSQC) 11

HSQC of 4-CeAcBu 11

HSQC of 6-CeAcBu-PAHE 12

IV. Fourier-transform infrared spectroscopy (FT-IR) spectra 13

V. UV-VIS spectra 14

VI. Solubility of novel components 15

I. ¹H and ³¹P NMR Spectra of synthesized components

Cellulose acrylate propionate ***(4-CeAcPr)***

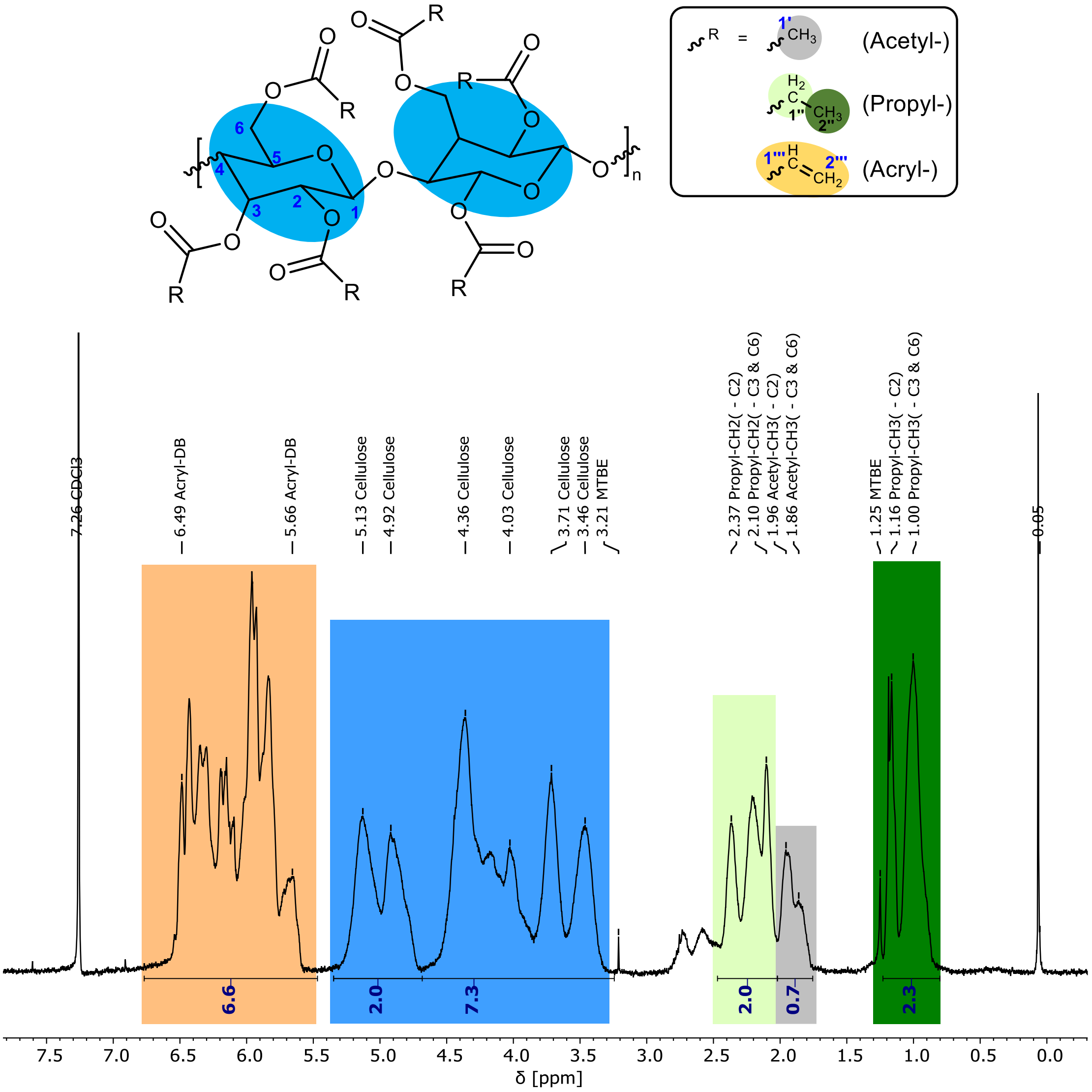

Figure S1. The functional groups are highlighted in colors according to their peak. ¹H NMR spectrum of cellulose acrylate propionate **(4-CeAcPr)** in $CDCl_3$ (300 MHz). Chloroform: 7.26 ppm (& 0.05 ppm impurity), MTBE: 3.21 ppm & 1.25 ppm.

Phosphorylated cellulose acrylate propionate with PAHE ***(6-CeAcPr-PAHE)***

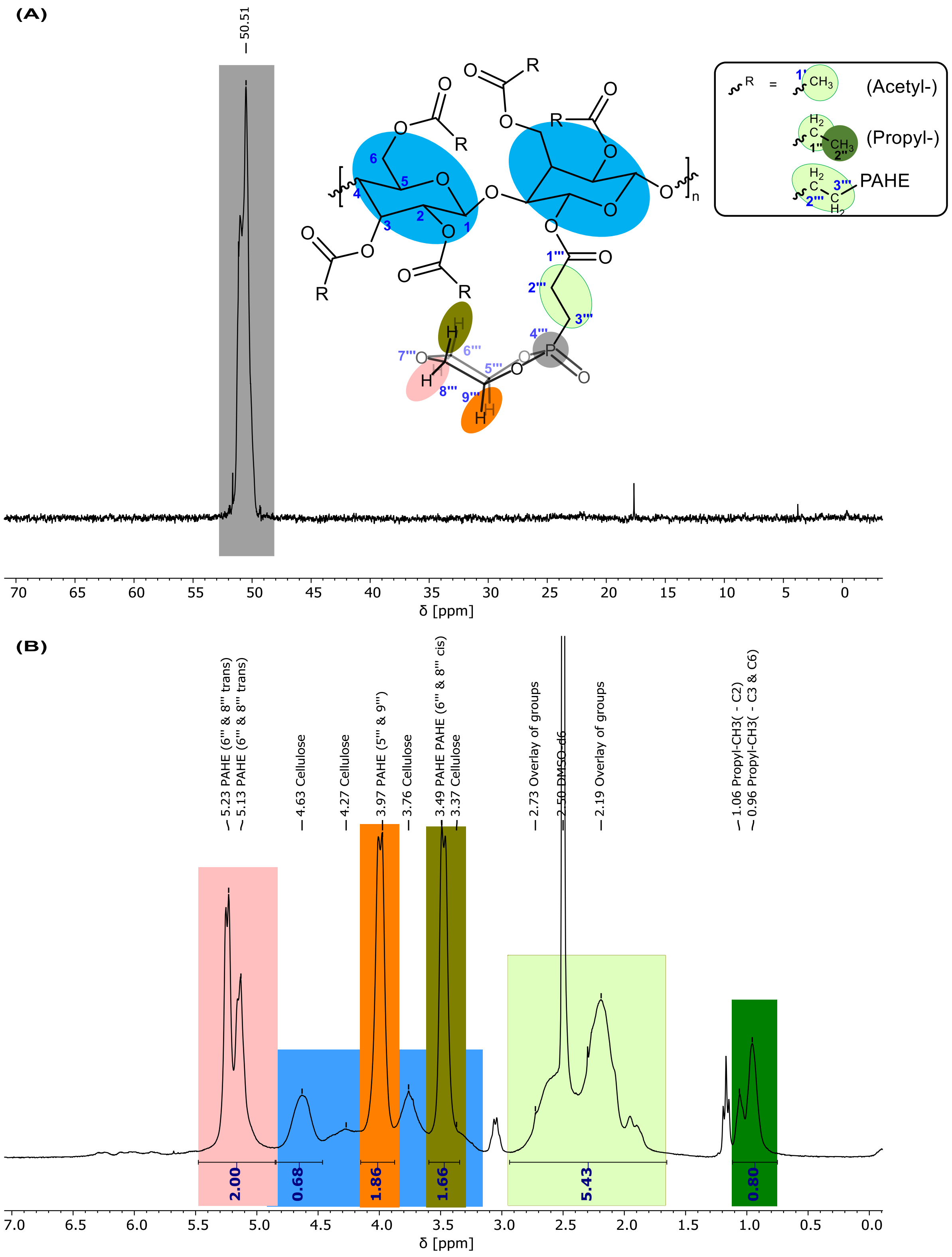

Figure S2. (A) ^{31}P NMR spectra of the PAHE functionalized cellulose acrylate propionate **(6-CeAcPr-PAHE)** in d6-DMSO (300 MHz). **(B)** ^{1}H NMR spectrum of the same (300 MHz). The functional groups are highlighted in colors according to their peak. The PAHE signals are overlapping with the cellulose signals as well as the overlay marked at 2.19 ppm (lime green). The latter contains acetyl-CH_3, propyl-CH_2 and the newly formed -CH_2-CH_2 group from the former acrylic double bond CH=CH_2. The spectrum shows still some impurities at 1.17 ppm and 3.04 ppm.

*Cellulose acrylate propionate (**4-CeAcPr**^{SA})*

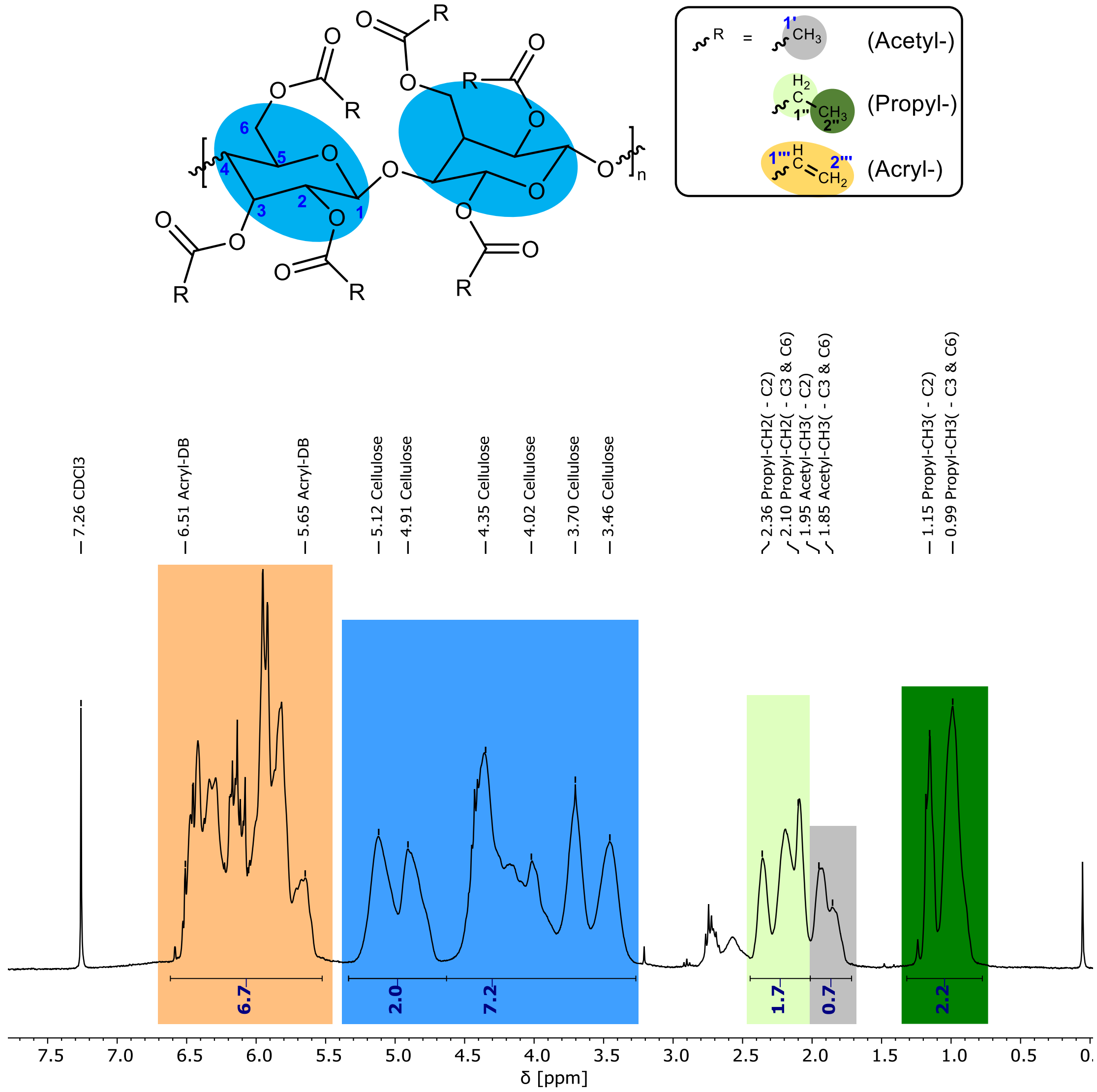

Figure S3. The functional groups are highlighted in colors according to their peak. ^{1}H NMR spectrum of cellulose acrylate propionate **(4-CeAcPr^{SA})** in $CDCl_3$ (300 MHz). Chloroform: 7.26 ppm (& 0.05 ppm impurity), MTBE: 3.21 ppm & 1.25 ppm.

Phosphorylated cellulose acrylate propionate with PAHE ***(6-CeAcPr^{SA}-PAHE)***

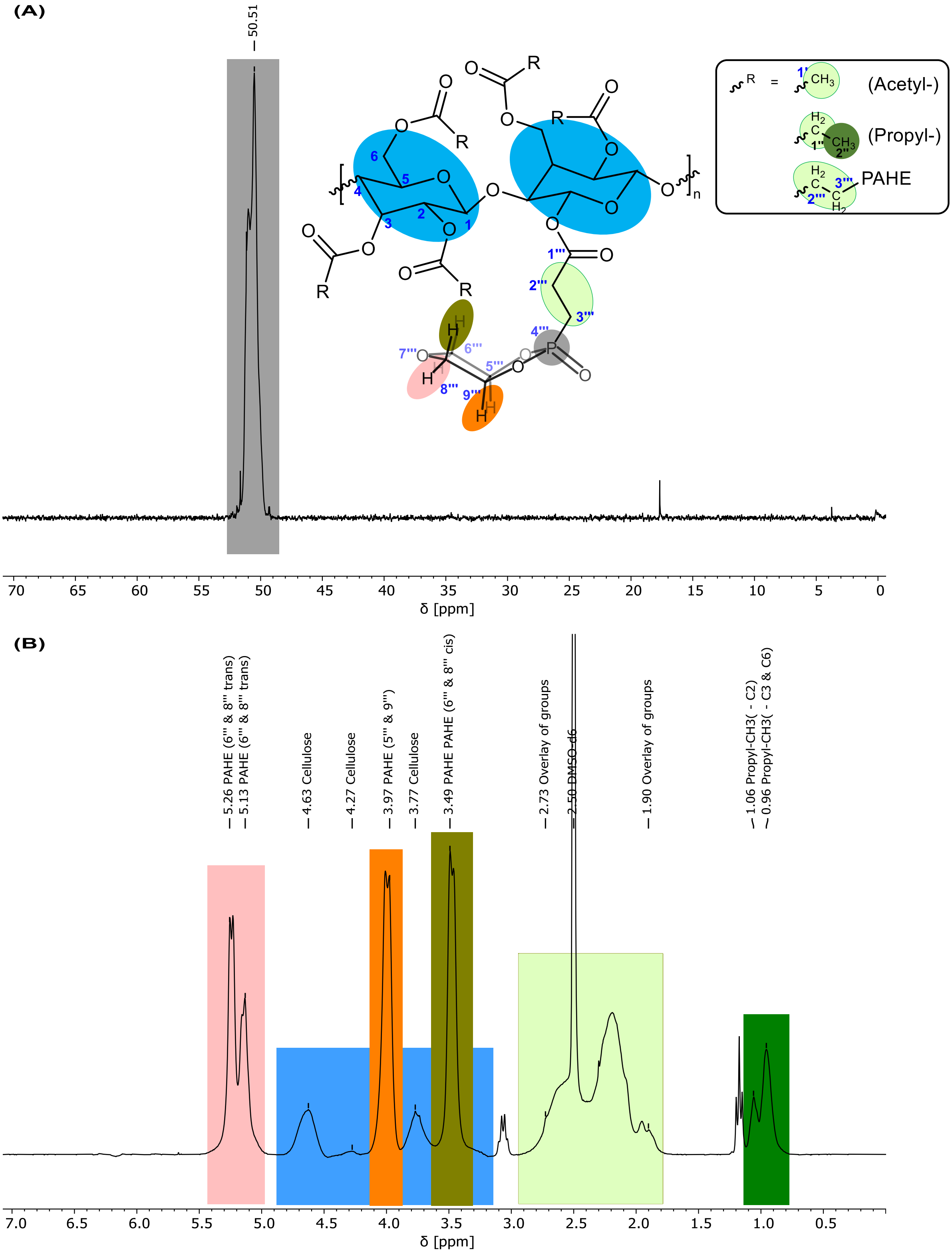

Figure S4. (A) ^{31}P NMR spectra of the PAHE functionalized cellulose acrylate propionate **(6-CeAcPr^{SA}-PAHE)** in d_6-DMSO (300 MHz). **(B)** ^{1}H NMR spectrum of the same (300 MHz). The functional groups are highlighted in colors according to their peak. The PAHE signals are overlapping with the cellulose signals as well as the overlay marked at 2.19 ppm (lime green). The latter contains acetyl-CH_3, propyl-CH_2 and the newly formed -CH_2-CH_2 group from the former acrylic double bond CH=CH_2. The spectrum shows still some impurities at 1.17 ppm and 3.04 ppm.

Phosphorylated cellulose acrylate propionate with DOPO ***(6-CeAcPr-DOPO)***

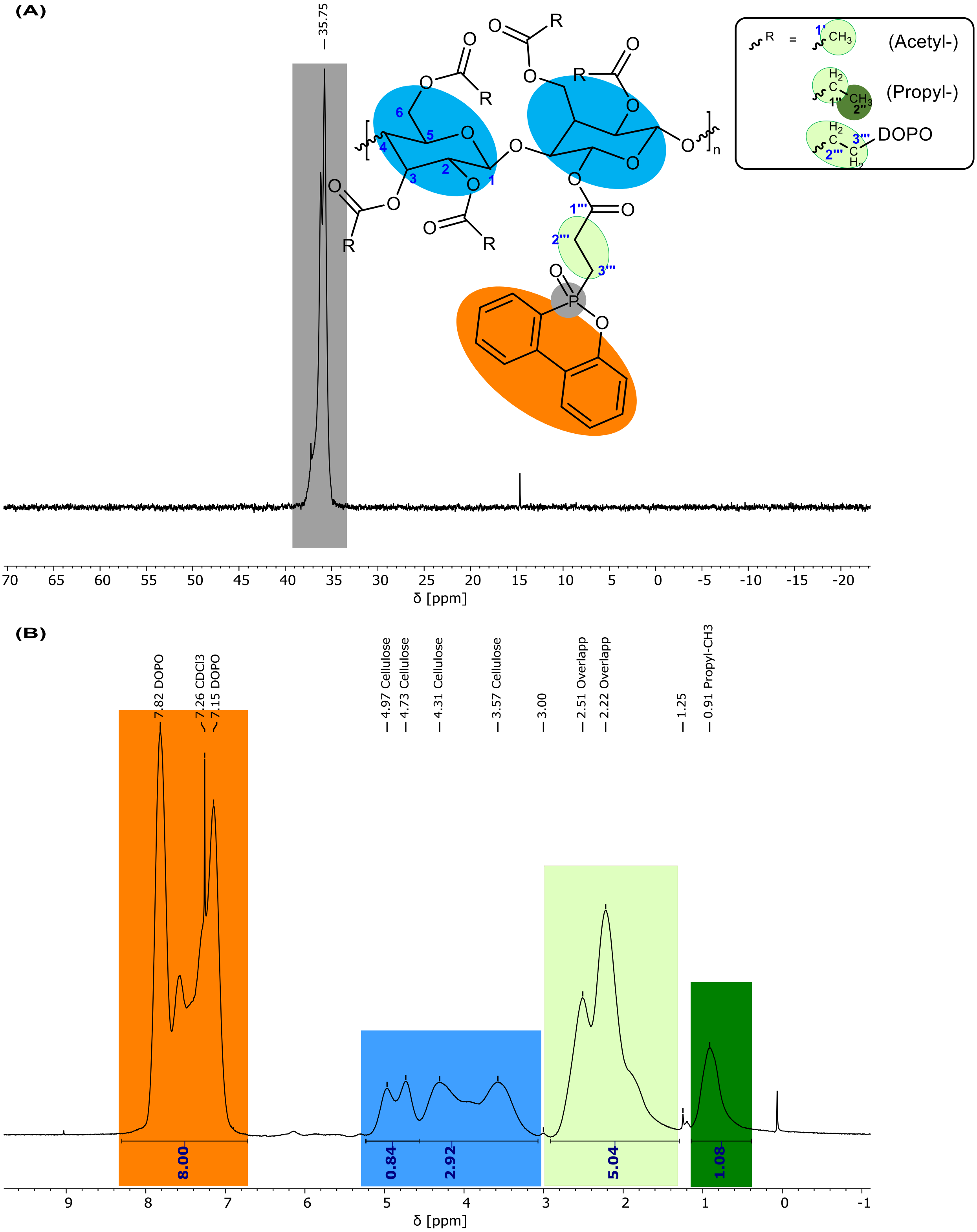

Figure S5. (A) ^{31}P NMR spectra of the DOPO functionalized cellulose acrylate propionate (**6-CeAcPr-DOPO**) in $CDCl_3$ (300 MHz). **(B)** ^{1}H NMR spectrum of the same (300 MHz). The functional groups are highlighted in colors according to their peak. The aromatic DOPO signals are clearly visible, while the overlay marked at 2.51 – 2.22 ppm (lime green) contains acetyl-CH_3, propyl-CH_2 and the newly formed -CH_2-CH_2 group from the former acrylic double bond -CH=CH_2. The spectrum shows still some impurities at 1.25 ppm and 3.00 ppm.

*Phosphorylated cellulose acrylate butyrate with DOPO **(6-CeAcBu-DOPO)***

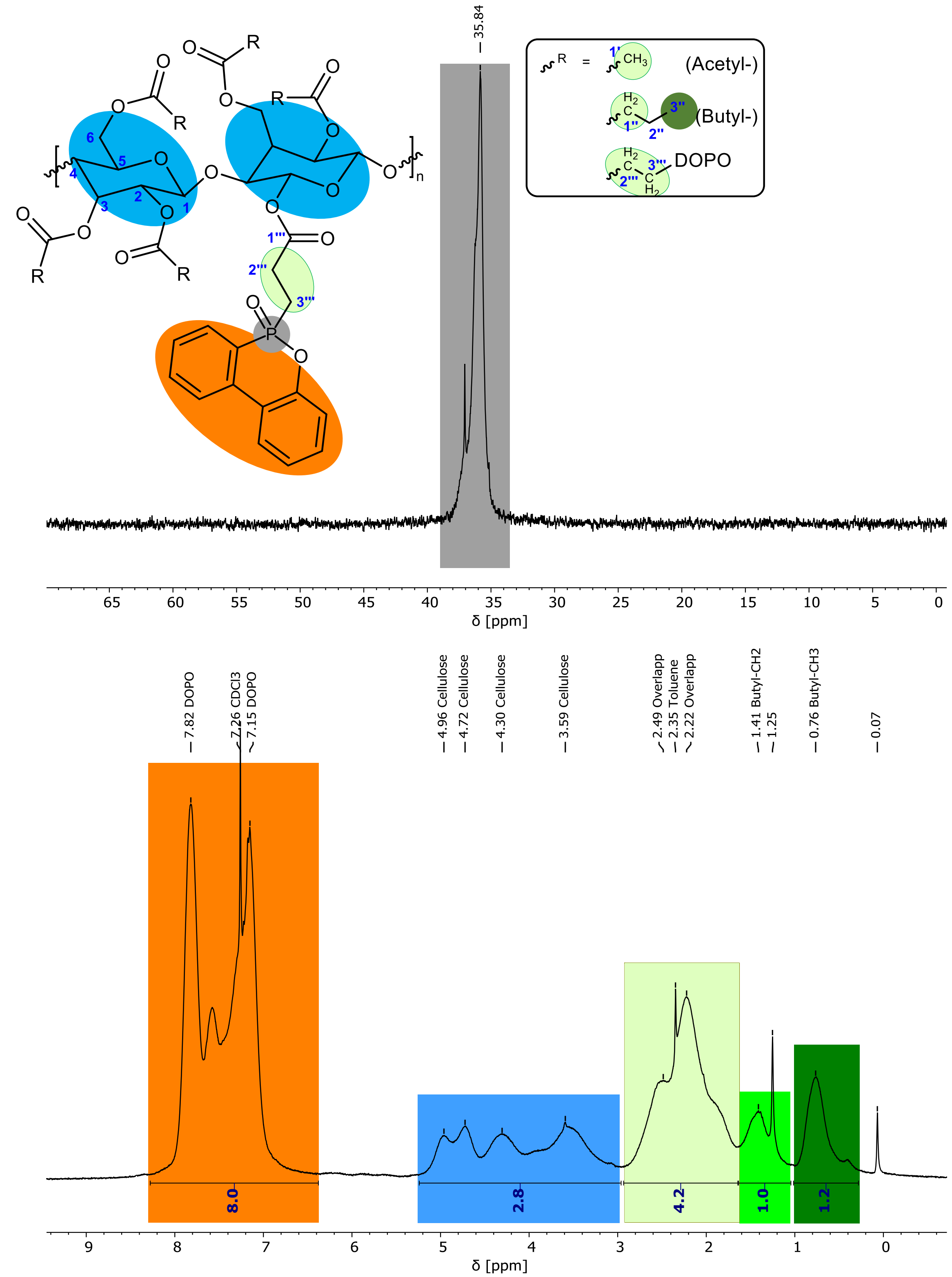

Figure S6. **(A)** ^{31}P NMR spectra of the DOPO functionalized cellulose acrylate butyrate **(6-CeAcBu-DOPO)** in $CDCl_3$ (300 MHz). **(B)** ^{1}H NMR spectrum of the same (300 MHz). The functional groups are highlighted in colors according to their peak. The aromatic DOPO signals are clearly visible, while the overlay marked at 2.51 – 2.25 ppm (lime green) contains acetyl-CH_3, propyl-CH_2 and the newly formed -CH_2-CH_2 group from the former acrylic double bond -CH=CH_2.

II. *Correlation spectroscopy (COSY)*

COSY of 4-CeAcBu

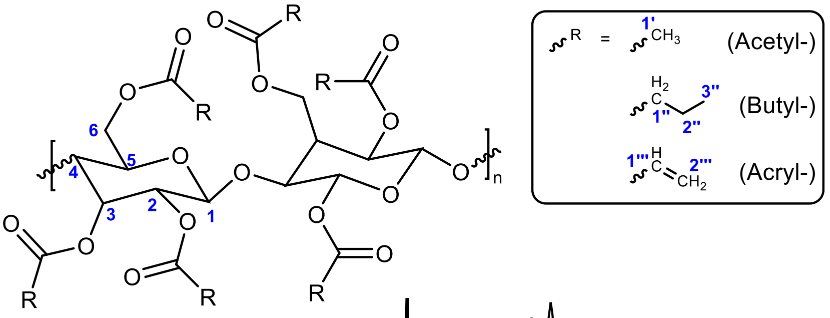

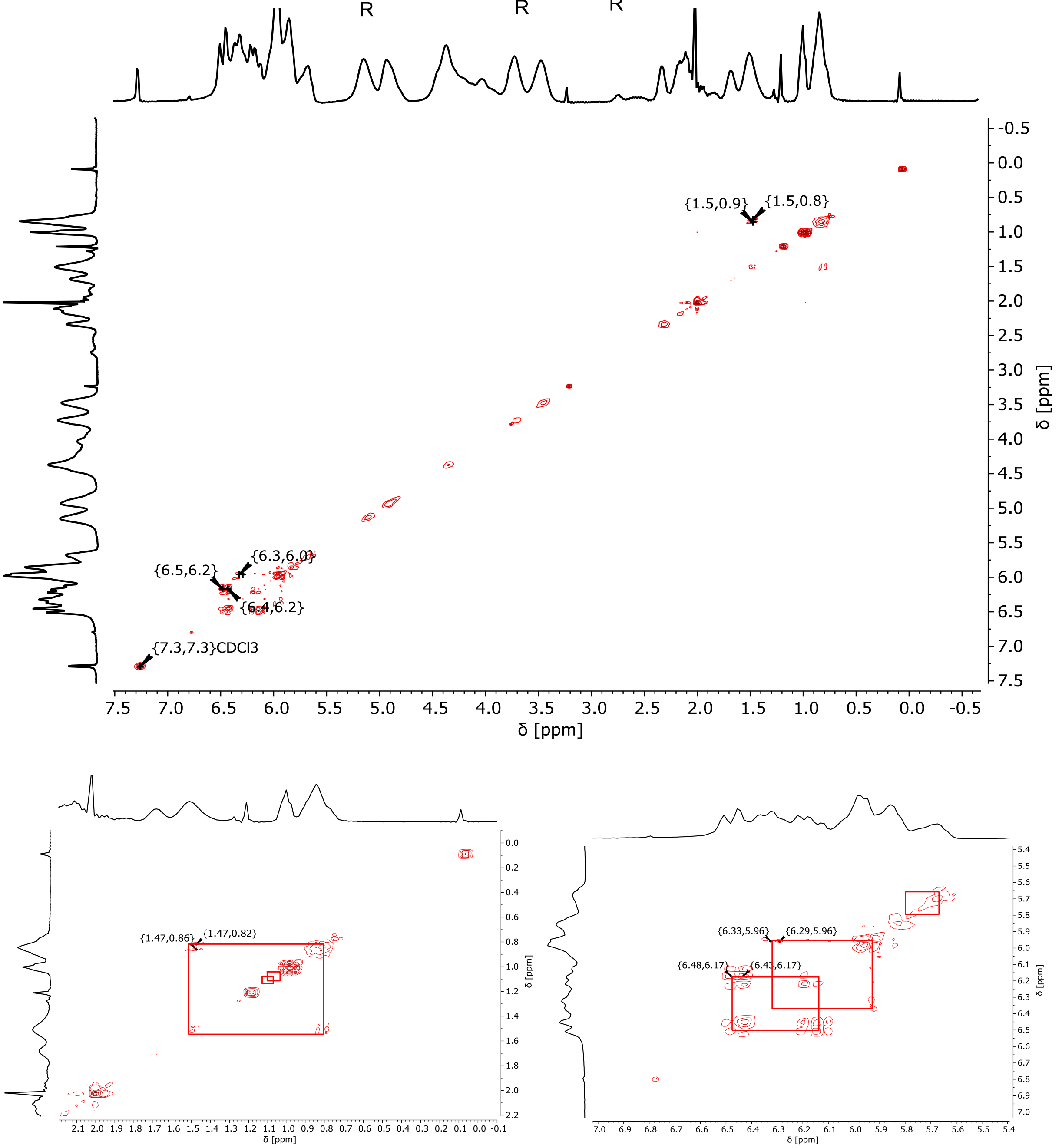

Figure S7. Correlation spectroscopy of cellulose acrylate butyrate (4-CeAcBu). Coupling patterns are highlighted as cutouts for **2′′-H** and **3′′-H** (coupling of two heterotopic protons of the butyl groups) and for **1′′′-H** and **2′′′-H** (coupling of three heterotopic protons of the acrylic protons). Not all coupling patterns are visible due to the limited resolution of the magnet field (300 MHz).

COSY of 6-CeAcBu-PAHE

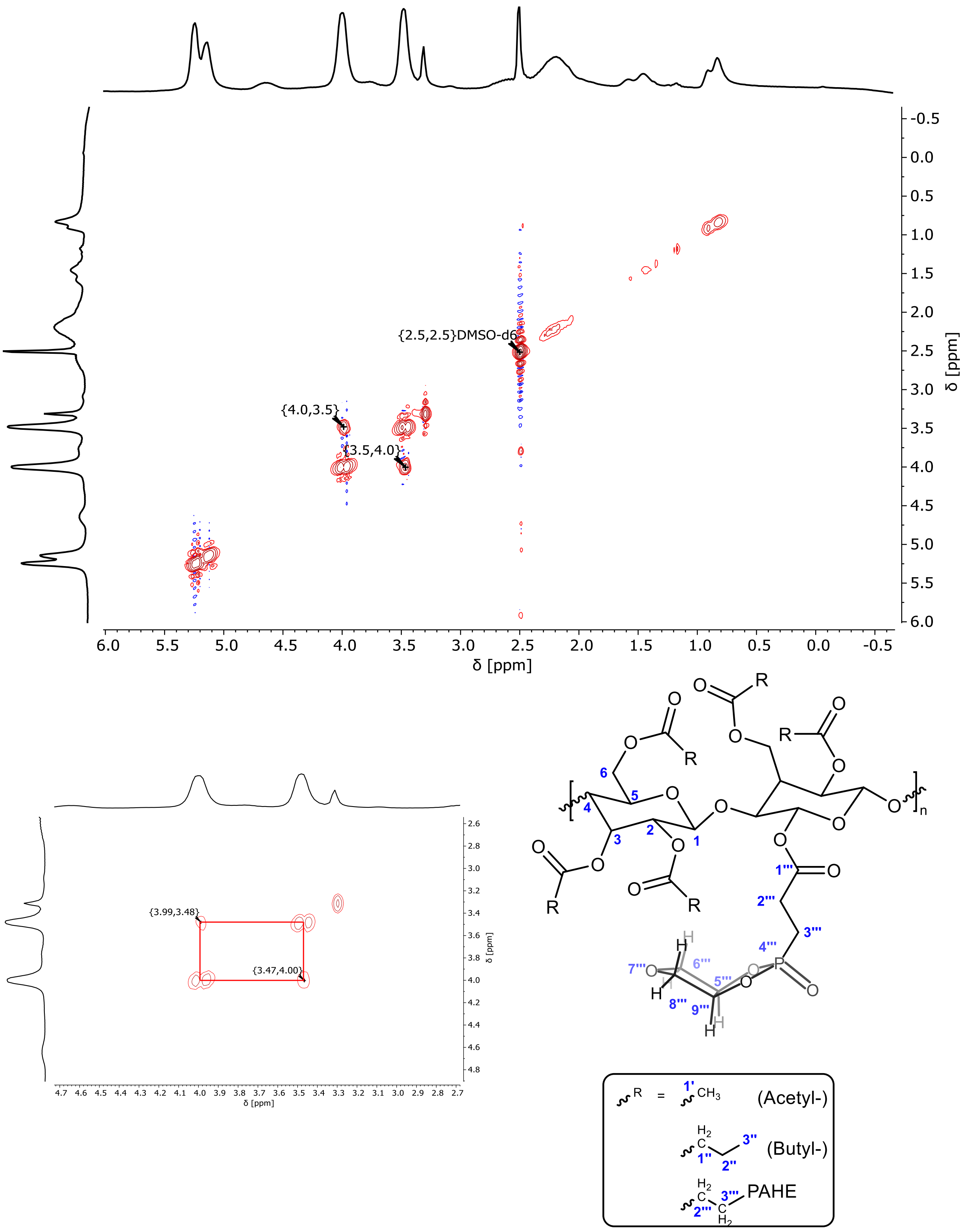

Figure S8. Correlation spectroscopy of phosphorylated cellulose acrylate butyrate (6-CeAcBu-PAHE). The coupling pattern is highlighted as cutout for the coupling between **5′′′-H and 9′′′-H** with **6′′′-H and 8′′′-H in trans position.**

III. *Heteronuclear single quantum coherence spectroscopy (HSQC)*

HSQC of 4-CeAcBu

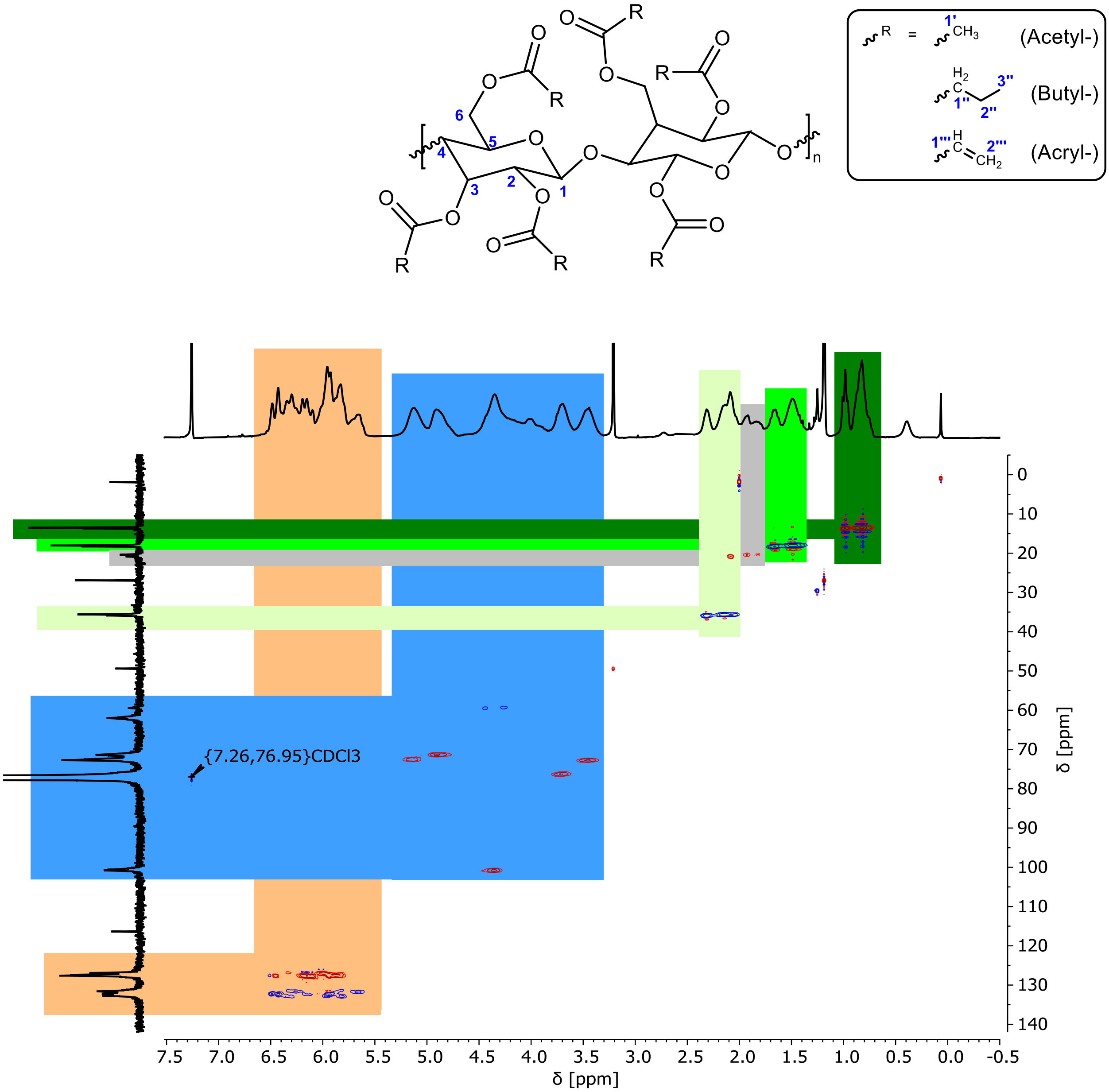

Figure S9. Heteronuclear single quantum coherence spectroscopy (HSQC) of cellulose acrylate butyrate (4-CeAcBu). With the aid of the HSQC spectra the peak assignment of the different proton/carbon atoms is supported. Unfortunately, not all cellulose atoms are visible due to the low magnet field force (300 MHz).

HSQC of 6-CeAcBu-PAHE

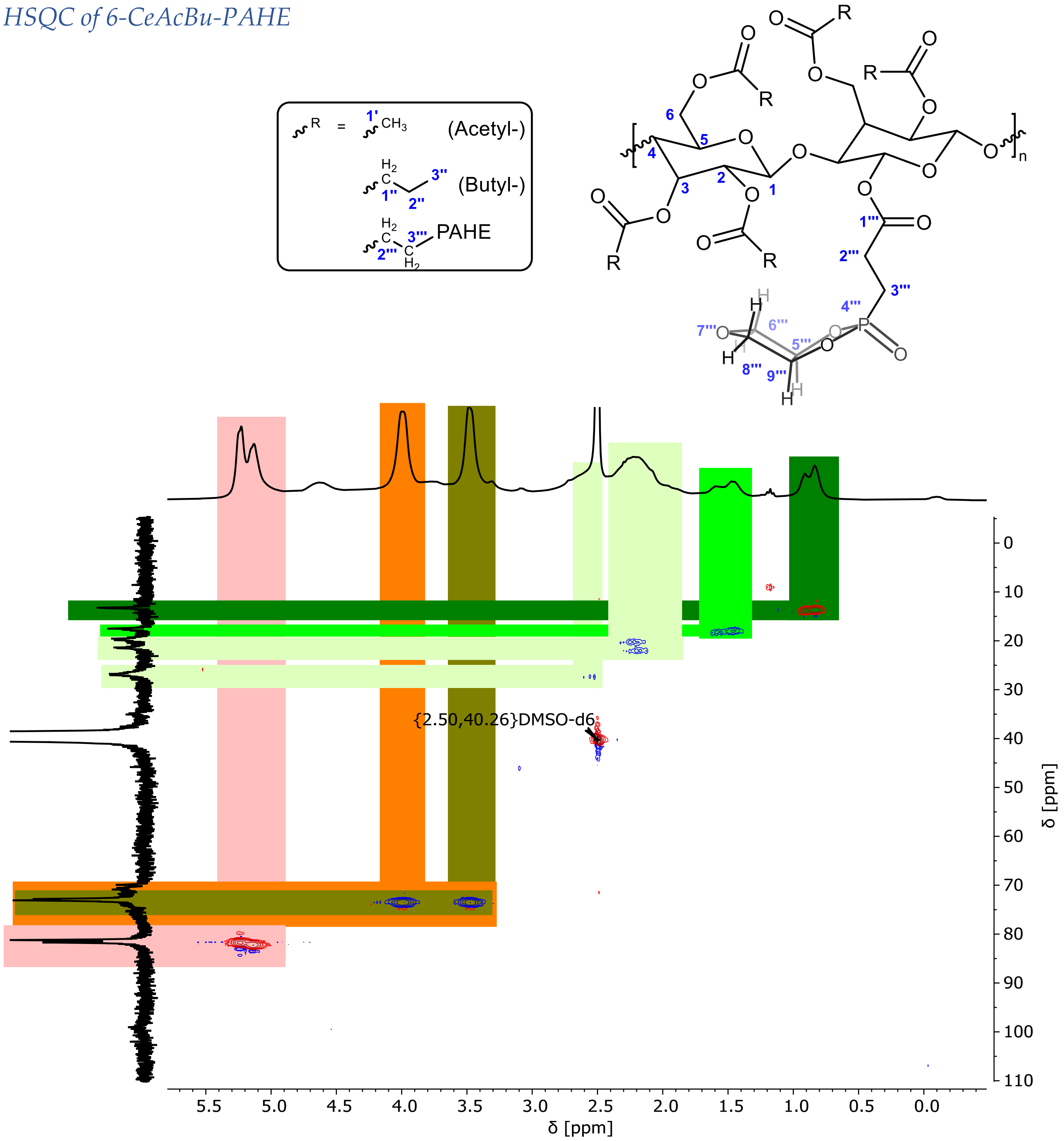

Figure S 10. Heteronuclear single quantum coherence spectroscopy (HSQC) of phosphorylated cellulose acrylate butyrate (4-CeAcBu-PAHE). With the aid of the HSQC spectra the peak assignment of the different proton/carbon atoms is supported. Unfortunately, not none of the cellulose atoms are visible due to the low magnet field force (300 MHz).

IV. *Fourier-transform infrared spectroscopy (FT-IR) spectra*

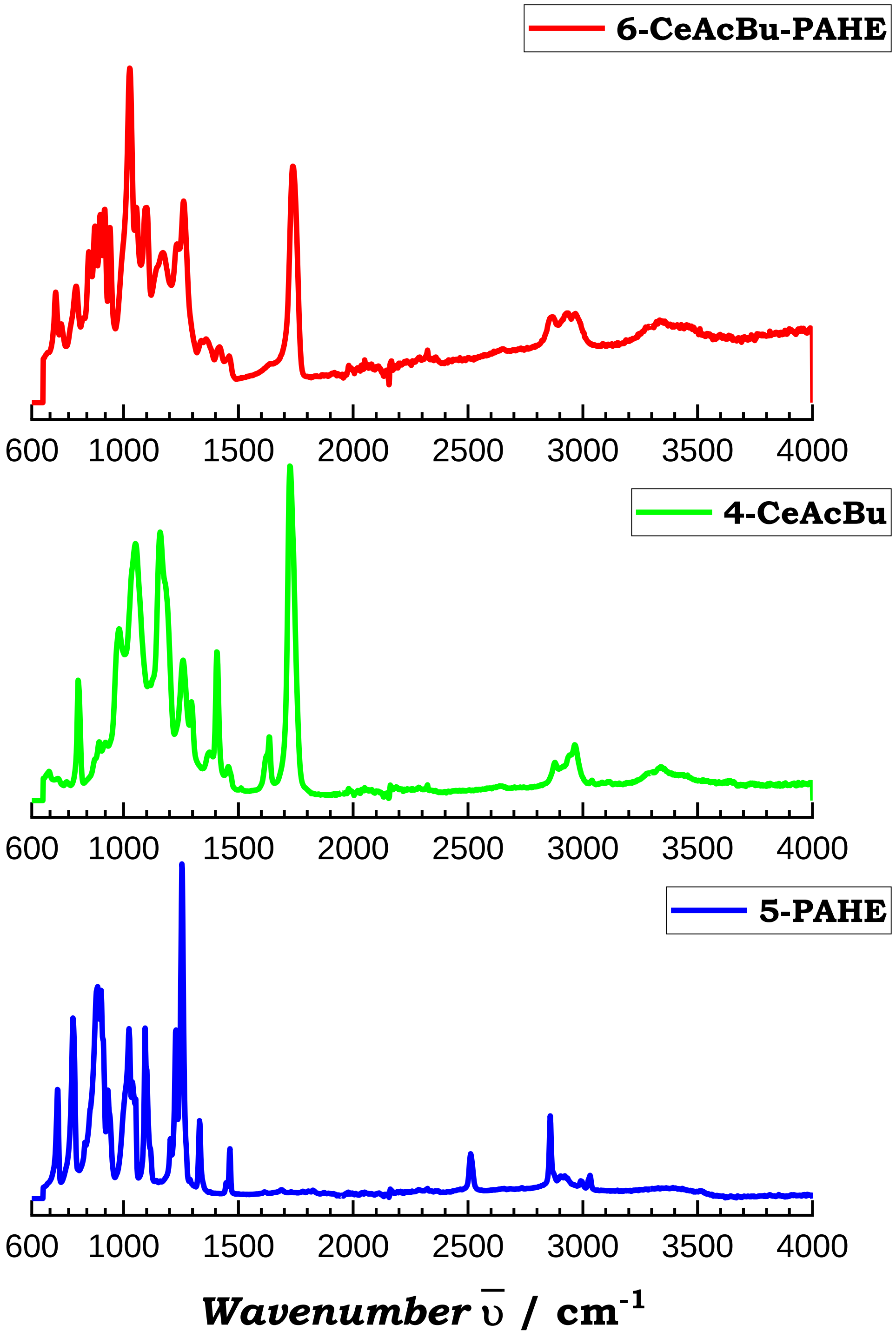

Figure S 11. Fourier-transform infrared spectroscopy (FT-IR) spectra of phosphorylated anhydro erythritole (PAHE), cellulose acrylate butyrate (4-CeAcBu) and phosphorylated cellulose acrylate butyrate (6-CeAcBu-PAHE). The characteristic C=C signal at ~ 1600 cm^{-1} and the acrylic C=O signal at ~ 1400 cm^{-1} vanishes after the phospha-michael addition.

V. UV-VIS spectra

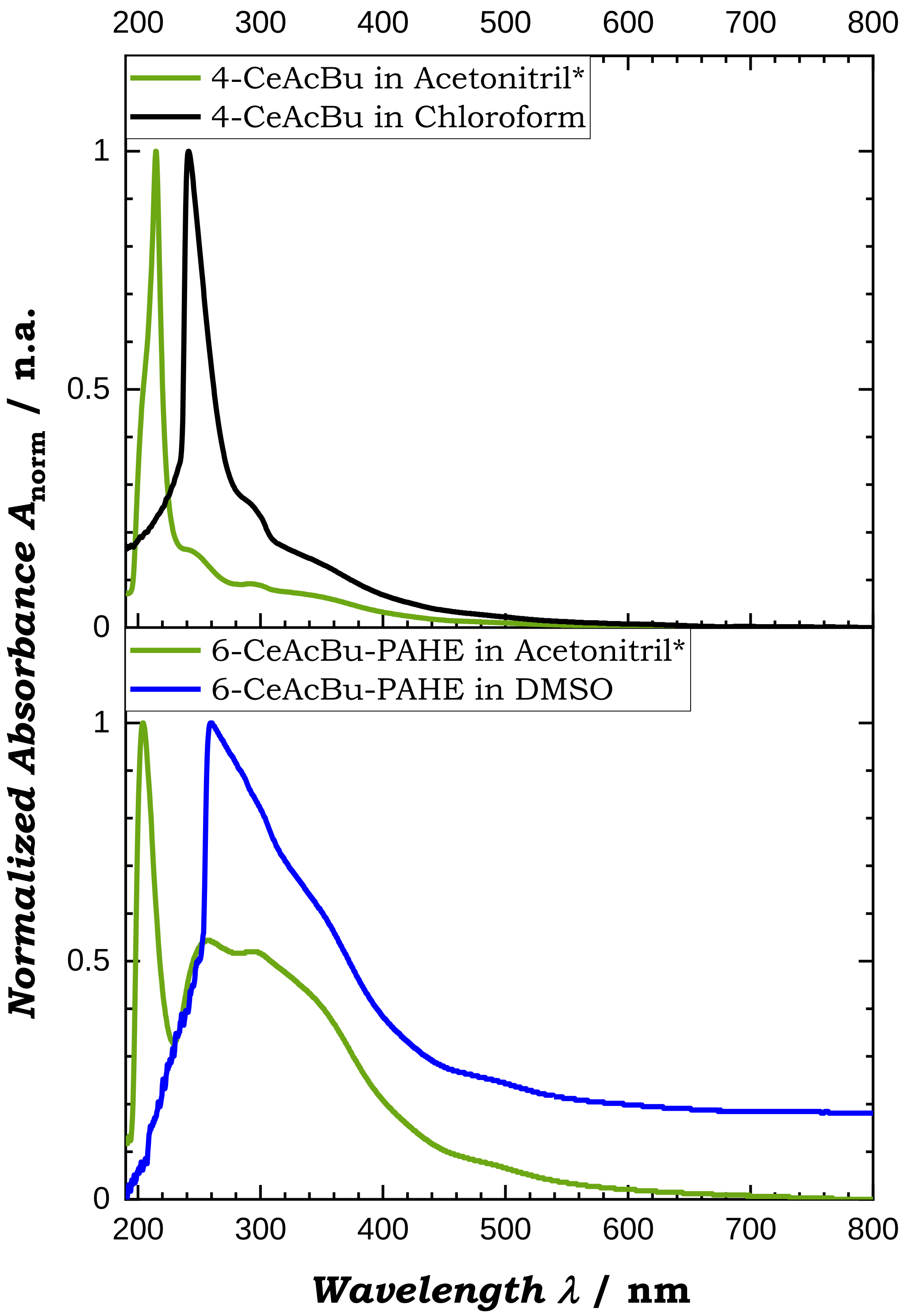

Figure S 12. UV-VIS spectra of cellulose acrylate butyrate (4-CeAcBu) and the phosphorylated derivate (6-CeAcBu-PAHE). Due to the low solubility in acetonitrile, the measurements were also performed in a good solvent (chloroform and dimethyl sulfoxide), which led to signal overlapping.

VI. Solubility of novel components

Table S1. Solubility of the novel components 4-CeAcBu (cellulose acrylate butyrate) and 6-CeAcBu-PAHE (phosphorylated cellulose acrylate butyrate).

Solvent	4-CeAcBu concentration [mg/mL]	6-CeAcBu-PAHE concentration [mg/mL]
Cylohexane	-	-
Diethyl ether	-	-
Chloroform	> 2	-
Acetone	1	-
Tetrahydrofuran	> 2	-
Methyl tert-butyl ether	-	-
Dimethyl sulfoxide	> 2	> 2
Acetonitrile	1	-
Pyridine	> 2	> 2
Water	-	1*

*Hydrolysis of phosphacycle likely
